# 

**Published:** 1910-07-09

**Authors:** 


### Appointments.

R. T. Slinger, M.B., Ch.B., F.R.C.S., has been appointed Honorary Anæsthetist and Surgical Pathologist to the Worcester General Infirmary.

H. Neville Crowe, M.B., Ch.B., M.R.C.S., L.R.C.P., has been appointed Honorary Anæsthetist and Medical Pathologist to the Worcester Royal Infirmary.

Dr. F. L. Golla has taken up his duties as physician to the Out-patient Department of the West End Hospital for Diseases of the Nervous System. He will attend on Friday evenings at 5.30.

The following appointments have been made by the Board of Management of the Manchester Royal Infirmary: W. P. H. Parker, L.R.C.P. and S.I., Assistant Medical Officer to the Barnes Convalescent Home, Cheadle; J. A. Jones, L.S.A., House Physician, Royal Infirmary; Dr. W. B. Anderton, re-appointed as Pathological Registrar; Howard Buck, F.R.C.S., re-appointed as Surgical Registrar; W. R. Douglas, F.R.C.S., appointed Assistant Surgical Officer; J. B. Macalpine, M.B., Ch.B. (Vict.), M.R.C.S., L.R.C.P., Medical Officer at the Central Branch.

### COMING EVENTS OF THE WEEK.

[*Announcements for this column must be sent in before* 1 *o'clock on Wednesday of the week preceding the meeting.*]

SUNDAY, JULY 10.—Visiting Day at the Hospitals.

MONDAY, JULY 11.—Annual Meeting, East End Mothers' Lying-in Home, Mansion House, 3 P.M., Lord Mayor in the chair.

TUESDAY, JULY 12.—Mr. Stephen Coleridge on "The Methods of the Research Defence Society," Kensington Town Hall, 8.30 P.M.

WEDNESDAY, JULY 13.—League of Mercy Meetings. Colchester District: Mrs. Lefroy, At Home, The Lodge, Boxted, Colchester, at 3 P.M.

West Marylebone District: Mr. and Mrs. Harold Hardy, At Home, 50 Great Cumberland Place, W., 4.30-6 P.M.

Holborn District: The Lady President, At Home, 37 Russell Square, W.C., at 4 P.M.

British Postal Medical Officers' Association: Dinner, Whitehall Rooms, Hôtel Métropole.

THURSDAY, JULY 14.—Foundation-stone Laying of Barnato-Joel Charity, which is to be established in connection with the Cancer Charity of the Middlesex Hospital.

FRIDAY, JULY 15.—Viscountess Portman's Garden Party in Aid of the Royal Waterloo Infants' Hospital.

Special *Matinée*, His Majesty's Theatre, in Aid of Royal Waterloo Infants' Hospital.

SATURDAY, JULY 16.—Provincial Sessional Meeting, Royal Sanitary Institute, Cambridge, 11 A.M.

### Gifts and Bequests.

Mr. Harry Mouel Hudson, of Streatham, S.W., has left £200 to King Edward's Hospital Fund.

The Infants' Hospital, Vincent Square, Westminster, has received £500 from Mrs. Ludwig Mond to endow the Arnold Cot.

The late Bishop of Lincoln (Dr. Edward King), who left estate of the gross value of £24,069, with net personalty £23,634, has left £500 to the Lincoln County Hospital.

The Leicester Infirmary has received £100 from the executors of the late Mrs. Mary Sparrow, of Blaby, and intimation that the late Mr. William Wilford, of Market Harborough, has bequeathed £20.

Prince Francis of Teck acknowledges £500 from Messrs. Baring Brothers and Co., £100 from Mr. A. Pearce Gould, and £100 from Messrs. Glyn, Mills, Currie and Co.; "Anonymous, King's Birthday," £100; Messrs. Panmure Gordon and Co., £105; and Messrs. A. Cartier and Son, £200, in response to his appeal on behalf of the Middlesex Hospital.

Mr. Isaac Bugg Coaks, of Norwich, has bequeathed £250 to the Norfolk and Norwich Hospital, and £100 each to the Jenny Lind Infirmary for Sick Children, Norwich, and the Norwich Society for Relieving the Sick Poor at their own Houses.

Mr. Thomas Galpin, of Palace House, Kensington Gardens, and of Clun House, Surrey Street, Strand, for many years managing director of Cassell and Co., Limited, has left 100 guineas each to the Homœopathic Hospital, Great Ormond Street, St. Thomas's Hospital, and the Dorset County Hospital.

Miss Ellen Elizabeth Bond, of Lancaster, subject to certain legacies, has left the residue of her property, apparently over £10,000, upon trust to erect a sanatorium for consumptives, to be built, if possible and desirable, near Lancaster, and on which residents of Lancaster shall have the first claim.

Mrs. Julia Scaramanga, of Hyde Park Gardens, has bequeathed £5,000 each to the General Hospital, Scio, and the Leper Island, Island of Scio, Greece; £200 to the Hospital for Consumption and Diseases of the Chest. She directed that all her medical books and all books in the Greek language are to go to the public library at Athens.

### Jottings.

The King and Queen have become patrons of the Brompton Hospital for consumption.

Queen Alexandra has sent a gift of chocolates to the Hospital for Sick Children, Great Ormond Street, W.C.

The Lord Mayor, as treasurer of the Hospital Sunday Fund, had received at the end of last week about £39,000 this year.

The new operation theatre at the Whitworth Hospital, Darley Dale, Notts, has been opened by Dr. Thorburn, of Manchester. The theatre cost £1,300.

Sir Savile Crossley announced at the quarterly meeting of the board of delegates last Saturday that the King has been graciously pleased to become patron of the Hospital Saturday Fund.

The National Provincial Bank of England has made a grant of £105 to the reconstruction scheme of the Leicester Infirmary through Mr. P. Campbell, the manager of the Leicester Branch of that Bank.

The Herefordshire County Council and the Hereford City Council have decided not to build a county sanatorium for consumptives, but to subsidise one if privately erected by contributing £600, to be repaid in bed accommodation. They consider a private hospital would be more economically erected and economically managed.

The thirty-eighth annual meeting of the governors of the Provident Surgical Appliance Society was held last week in Finsbury Circus, Mr. C. King presiding. In asking for increased support, the committee pointed out that the society did not receive grants from any of the hospital funds, although it was a great help to them.

The following ladies and gentlemen have been appointed house visitors for the next three months at the York County Hospital: Mrs. Guy S. Palmes, Mrs. M. H. Smith, Mrs. Cudworth, Miss Lloyd, Miss Coates, Mrs. C. A. Cooper, Sir J. Grant Lawson, Bart., the Rev. Canon Owen, the Rev. M. H. Smith, Mr. H. Precious, Mr. James Pollard, and Mr. J. H. Baldwin.

The King, Sovereign head and patron of the Order of the Hospital of St. John of Jerusalem in England, has become the patron of the British Ophthalmic Hospital at Jerusalem (belonging to the Order), in succession to King Edward VII. The Duke of Connaught, Grand Prior of the Order, has accepted the office of President of its ambulance department, the St. John Ambulance Association.

The National Naval Cadets will give a display at the Belgrave Hospital for Children, Clapham Road, S.W., on Saturday, July 9, at 3.30 p.m., in aid of the hospital, which is in urgent need of funds. Admiral the Hon. Sir E. R. Fremantle, a vice-president of the corps, will inspect the cadets, whose display will include field-gun drill, rifle exercises, and cutlass drill. The Cadets' brass band will be in attendance.

Lady Exeter a few days ago opened a Country Sanatorium at Creaton, Northamptonshire. Lord Northampton, President of the Northamptonshire branch of the Association for the Prevention of Consumption, presided, and Sir Frederick Treves gave an address on the prevention of consumption, strongly advocating sanatoria as a means by which 74 per cent. of the cases could be cured. The new sanatorium provides for twenty-two patients, and it is prophesied that it will soon have to be enlarged.

At a special meeting of the Board of Governors of the Bolingbroke Hospital, Wandsworth Common, held last week, the following resolution was passed: "That having regard to the judgment of the Court of Appeal wherein the gift of £5,000 from the Weir Trust Funds to the hospital was described as a misapplication of such fund, it is the unanimous desire of the Governors to restore such sum, if possible; and that it will be referred to a committee to consider and report, after taking legal advice, the best steps to be taken to give effect to such desire."

### TO HOSPITAL SECRETARIES AND OTHERS.

We invite contributions from any of our readers, and shall be glad to pay, according to length, for items of news for the institutional section (including appointments, resignations, gifts, etc.) which we may publish. All payments for manuscripts used are made as early as possible after the beginning of each quarter.

THE HOSPITAL. July 9, 1910.

*Name* ..........................................

*Address* ..........................................

..........................................

This Coupon must accompany manuscript or contributions intended for The Hospital.

The annual meeting of the Orsett Joint Hospital Board was held at the Hospital, Stifford, on Monday last. Mr. T. N. Banks has been re-elected chairman and the Rev. G. J. Llewelyn vice-chairman (in the place of Mr. F. Kemp-Smith, who recently retired) of the Orsett Joint Hospital Board.

**The Institution Library.**

"Burdett's Hospitals and Charities," 1910. (Net. post free.) ... ... ... ... ... ... ...10s. 11d.

"Hospital Expenditure: The Commissariat." For the use of Managers. ... ... ... ... ... 2s. 6d.

"The District Nursing Association; Hints on how to Start it." (Net, post free.) ... ... ... 1s. 1d.

"A Legal Handbook for the use of Hospital Authorities." ... ... ... ... ... ... ... 2s. 6d.

Ledgers, Charts, Case-papers, and every kind of Institutional Literature.

Of all booksellers or of The Scientific Press, Limited, 28 and 29 Southampton Street, Strand, London, W.C.

